# Supplementary material for: An adjustable algal chloroplast plug-and-play model for genome-scale metabolic models
Source: PLoS One. 2020 Feb 24;15(2):e0229408. doi: 10.1371/journal.pone.0229408 (PMC7039451; doi:10.1371/journal.pone.0229408)
Supplement: S1 Text — Detailed description of the modelling tools included in S2 File. (DOCX) [file pone.0229408.s003.docx]

**S3 Text – Description of modelling tools**

Detailed descriptions of new scripts having been developed alongside with the iGR774 model.

**Scripts for adding reactions to a model**

- **addRxFromModelSyntaxGenerator**Generates syntax for adding a list of reactions taken from one model, to another model. The script translates names and IDs of the metabolites involved in the reactions to be transferred into the namespace of the model the reactions should be transferred to. The script also ensures that the reaction IDs and names are not already present in the model. If they are, the user is asked to give the reaction in question a new name and / or ID. The script also lets the user choose if the reaction should be organism-specific, and if it is, the lower and upper flux limits will be set accordingly. If *Nannochloropsis* is chosen, flux limits will be 0 and 1000 for an irreversible reaction and -1000 and 1000 for a reversible reaction. If the reaction should be part of the *Chlamydomonas* or *Phaeodactylum* specific parts of the model, both the lower and the upper flux limit is set to 0, as *Nannochloropsis* mode is considered the default mode of the model.
  Inputs:
  - List of reactions numbers (numbers referring to the correct reaction indexes in the model the reactions should be transferred from)
  - Model reactions should be transferred from
  - Model reactions should be transferred to

Outputs:

- - Syntax for adding the reactions to the model they should be transferred to. Syntax for adding reactions is generated both for addReaction (included in COBRA Toolbox), and addRxInfo, which is described below.
- **pickKEGGrx**Generates syntax for adding a reaction from KEGG to a model. The metabolites are compared to the metabolites already included in the model on the basis of KEGG ID, and if they are already present, the metabolite name and ID from the model is adopted. If one or more metabolites is not present in the model, the user is asked to define names and IDs. The script suggests the metabolite names from KEGG, but lets the user choose. The chosen metabolite ID is checked against the model to make sure a metabolite with the same ID does not already exist.
  The script lets the user choose if the reaction should be reversible or not, and also if it should run in the same direction as it is defined in KEGG, or in the opposite direction.
  The script lets the user choose if the reaction should be part of an organism-specific mode, and sets lower and upper flux limits accordingly.
  Inputs:
  - Model the reaction will be transferred to
  - KEGG ID of reaction (on the format RXXXXX, where X is a number between 0 and 9)
  - Directionality of reaction (1 if the user wants to run the reaction in the same direction as it is defined in KEGG, -1 if the opposite direction is preferred)

Outputs:

- - Syntax for adding KEGG reaction to a specific model. Syntax for both addReaction (included in COBRA Toolbox), and addRxInfo (described below) is generated.
- **rxGenerator**Generates syntax for adding a reaction specified by the user to a model. The user can search the model for metabolites, add new metabolites, or choose already existing metabolites to a new compartment.
  The script lets the user specify stoichiometric coefficients for the metabolites involved in the reaction, in addition to reaction ID (which is checked against the model.rxn vector to make sure it is not already present), reaction name, reversibility (which is used to set flux limits), sub system, EC number, and reaction reference.
  Input:
  - Model structure

Outputs:

- - Syntax for adding reaction to the model. Syntax both for addReaction (included in COBRA Toolbox), and addRxInfo (described below) is generated.

**Scripts for adding genetic information about a certain reaction in a model**

- **addGenes**Adding genetic information for a specific reaction to a model. The script adds the genes to model.genes (if not already present). The genes are also associated with the reaction in model.rxnGeneMat, and entries for rules and grRules are generated.
  Inputs:
  - Model structure
  - Reaction ID
  - List of genes
  - And / or relationships (optional. If and/or relationships are not given as an input, the user will instead be asked by the script if an AND or an OR relationship should be used for the genes associated with the reaction in question)

Output:

- - Model structure which updated genetic information for the reaction in question.
- **addRxInfo**Adds additional information to a reaction that is not added by the addReaction script included in COBRA Toolbox.
  Inputs:
  - Model structure
  - List of metabolite IDs of metabolites involved in reaction (used for identification of reaction, and for finding index of metabolites in model.mets vector)
  - List of KEGG IDs of metabolites involved in reaction (Not added by addReaction)
  - List of metabolite names of metabolites involved in reaction (Should be added by addReaction, but for some reason is usually not)
  - Name of reaction (Not added by addReaction)
  - Reaction references (Not added by addReaction) (optional input)
  - EC number (Not added by addReaction) (optional input)
  - List of genes associated with reaction (will be used to update genetic information in model.rxnGeneMat, and to generate rules and grRules. The user will be asked about and/or relationship between genes if gene list contains more than one gene) (optional input)

Output:

- - Model structure with updated metabolite names, metabolite KEGG IDs, reaction name, reaction reference, EC number and genetic information (adding new genes to model.genes, altering model.rxnGeneMat and creating rules and grRules) for specific reaction.

**Inspecting model in MATLAB and cytoscape**

- **matlab2cytoscape**Generates cytoscape importable .csv file from model structure. Both metabolites and reactions will be made nodes when the network is imported to cytoscape, while the links indicate a connection between a certain metabolite and a certain reaction. Information about the relationship between metabolite and reaction (reversible / irreversible) is exported alongside with the reaction – metabolite associations, and can be used as a link attribute. If present, the reaction flux vector generated by optimizeCbModel (included in COBRA Toolbox) can also be used as a link attribute.
  Inputs:
  - Model structure
  - The desired filename for the output .csv file
  - FBA optimization result generated by optimizeCbModel (optional)

Output:

- - .csv file for cytoscape import
- **pathway2cytoscape**Generates cytoscape importable .csv file from part of a metabolic model in the same way as for an entire network (matlab2cytoscape, described above). The relationship between metabolite and reaction (reversible / irreversible) can be imported as a link attribute.
  Inputs:
  - Model structure
  - Reaction vector with numbers of reactions to be included in cytoscape network

Output:

- - .csv file for cytoscape import
- **metabolitePath**Lets the user inspect a path of metabolites connected by reactions. The script lets the user search for the start metabolite, and also state if this metabolite should be viewed as a product or a substrate (important for determining which reactions that can produce / consume metabolite, if reaction is irreversible). Each time the script is run, the script will identify which new metabolites the start metabolite can be created from / turned into, and the user gets to decide which metabolite she / he wants to monitor further. A flux vector from FBA optimization by optimizeCbModel can be imported (optional), and used to show the user the flux of the listed reactions.
  Inputs:
  - Model structure
  - FBA optimization generated by optimizeCbModel (optional)

Output

- - Text showing path from one metabolite to another (accompanied by flux of reaction if flux vector is present)

**Adding metabolite KEGG IDs to a metabolic model**

- **metKEGGIDsearch**Script with a high degree of automation running through the metabolites of a model and attempts to find their corresponding KEGG ID by searching for the metabolite names in KEGG. If a KEGG ID is identified for a certain metabolite, it is saved in model.metKEGGID.
  Input:
  - Model structure

Output:

- - Model structure with updated information about metabolite KEGG IDs
- **fillKEGGIDholes**More manual versjon of metKEGGIDsearch letting the user search in KEGG for the metabolites that could not be found in KEGG by the former script. metKEGGIDsearch and fillKEGGIDholes are meant to complement each other.
  Input:
  - Model structure

Output:

- - Model structure with updated information about metabolite KEGG IDs
- **KEGGIDfirstAid**Attempts to add KEGG IDs to a section of the metabolites in a model. Attempting to find KEGG IDs for *all* the metabolites present in a model by using metKEGGIDsearch and fillKEGGIDholes can be tedious work, despite metKEGGIDsearch’s high degree of automation, as metabolic models can sometimes contain several thousand metabolites. Yet, certain KEGG IDs has to be present if a sub-model is to be plugged into an exo-model. The metabolites that will interact between the two models to be merged are the metabolites that are present in the cytosolic compartment of the sub-model (if the sub-model is an organelle, the cytosolic compartment will be similar to the external compartment of a full cell model).
  KEGGIDfirstAid therefor attempts to find KEGG IDs only for the metabolites of the exo-model that will interact with the sub-model.
  Input:
  - Model structure for exo-model
  - Model structure for sub-model

Output:

- - Model structure for exo-model, with KEGG IDs added for the metabolites that will interact with the sub-model

**Scripts developed for working with organism-specific sub-models**

- **plugAndPlay**Script plugging a sub-model describing an organelle into an exo-model. The script works in several steps. First, the script saves backup versions of both models to be merged, as they will both be changed by the script. Then, the chloroplast biomass reaction is deleted from the chloroplast model, as the biomass reaction of the exo-model will be the prevailing biomass reaction when the two models are merged.
  The script also checks that the compartment symbols of the two models are on a similar format.
  Before merging the models, the script checks the amount of metabolites with an associated KEGG ID, and if the percentage is low, lets the user attempt to search for KEGG IDs either by using metKEGGIDsearch and fillKEGGIDholes, or by using KEGGIDfirstAid.
  After filling in KEGG IDs, the exchange metabolites of the chloroplast model are translated into the exo-model namespace by comparing KEGG IDs for finding the corresponding metabolite in the exo-model.
  Subsequent to this step, the models are merged.
  The last step of the script is optional, and lets the user choose if she / he wants to adjust the exo-model biomass reaction to account for additional production of certain metabolites by the newly acquired chloroplast.
  Inputs:
  - Model structure of exo-model
  - Model structure of sub-model

Outputs:

- - Merged model structure
  - List of metabolites exported or imported by the sub-model, that are missing in the exo-model. If a metabolite used by the chloroplast sub-model is not present in the exo-model, this could cause the merged model not to run.
- **changeOrganismMode**Changes organism mode of a model by adjusting the upper and lower flux limits of the organism-specific reactions.
  If the objective function is an organism-specific reaction, this is also changed by the script, so the correct organism-specific biomass reaction will be set the objective function.
  Input:
  - Model structure

Output:

- - Model structure with changed upper and lower flux limits for organism-specific reactions (and a changed objective function, if the biomass reaction of the model in question happen to be organism-specific)

**Easing work of working with model in MATLAB**

- **dispEnzymeRx**Displaying information from KEGG about the reaction(s) catalysed by a certain enzyme. The script displays the KEGG ID of the reaction(s) the enzyme is responsible for catalysing, the name(s) of the reaction(s), and reaction equation(s) with metabolite names and KEGG IDs.
  Input:
  - EC-number of enzyme

Output

- - No output variables, but reaction info displayed on screen
- **dispFlux**Displays reaction information alongside with the fluxes from an FBA optimization.
  Input:
  - Model structure
  - Output structure from optimizeCbModel
  - List of reactions to be viewed (optional)

Output:

- - Reaction names, reaction equation and fluxes are displayed for all reactions or a subset of reactions
- **dispMet**Displays information about a particular metabolite in a model.
  Input:
  - Metabolite number in model

Output:

- - Information about metabolite ID, metabolite name and KEGG ID stored in model
- **dispRxns**Displays all reactions involving a certain metabolite. Lets the user search for the metabolite within the script.
  Input:
  - Model structure

Output

- - List of reactions involving the metabolite in question
- **KmetSearch**Searching for metabolites in KEGG, and displays all metabolite hits in KEGG that matches the text search string inputed by the user.
  Input:
  - Search word (metabolite name, or part of metabolite name)

Output:

- - Table with names and KEGG IDs of all metabolites matching search word
- **KEGGrxSearch**Displays all KEGG reactions involving one particular metabolite, or all reactions involving a particular set of metabolites. The latter option can be used if one for example wants to find all reactions in KEGG that involves both CO_2_, orthophosphate and Acetyl-CoA.
  For every KEGG reaction the script finds, KEGG reaction ID, EC number of enzyme catalysing reaction (if present), reaction equation written with metabolite names and metabolite KEGG IDs, and links to other databases (if present) is written out on screen.
  Input:
  - List of any number of metabolite KEGG IDs

Output:

- - List of reactions the input metabolites have in common. Additionally, info about all reactions in the output list is displayed on screen while the script is running
- **ReactionNames**
  Generates reaction equations with metabolite names for every reaction in a model.
  Input:
  - Model structure

Output:

- - List of reaction equations
