# Supplementary material for: An adjustable algal chloroplast plug-and-play model for genome-scale metabolic models
Source: PLoS One. 2020 Feb 24;15(2):e0229408. doi: 10.1371/journal.pone.0229408 (PMC7039451; doi:10.1371/journal.pone.0229408)
Supplement: S2 Text — Detailed composition of the protein, membrane lipids and pigment fractions included in the chloroplast biomass component. (DOCX) [file pone.0229408.s004.docx]

**S4 Text - Composition of chloroplast biomass components**

Detailed composition of chloroplast biomass components in the three organisms currently included in the chloroplast model.

The general chloroplast biomass reaction used for all three organisms is

*10 ATP + 5 H_2_O + 5 NADPH + 5 H^+^ + 2.5 O_2_ + 1 CPr + 1 CML + 1 CPi*

*→ 10 ADP + 10 P_i_ + 5 NADP^+^*

where CPr, CML and CPi denote ‘chloroplast proteins’, ‘chloroplast membrane lipids’ and ‘chloroplast pigments’, respectively. These three chloroplast biomass components are organism specific, and are described in detail below.

***Nannochloropsis***

*Nannochloropsis* chloroplast proteins

The amino acid composition for *Nannochloropsis* chloroplast proteins were taken from Xiao et al. 2013, and stoichiometric coefficients were set so the chloroplast model can create proteins consisting of 350 amino acids, which is the approximate length of an average algal protein.

| *Stoichiometric coefficient* | *Amino acid* |
| --- | --- |
| - 24.5 | Aspartic acid |
| - 38.5 | Glutamic acid |
| - 24.5 | Asparagine |
| - 38.5 | Glutamine |
| - 14.0 | Serine |
| - 14.0 | Threonine |
| - 17.5 | Arginine |
| - 7.0 | Histidine |
| - 17.5 | Lysine |
| - 17.5 | Glycine |
| - 3.5 | Cysteine |
| - 14.0 | Proline |
| - 24.5 | Alanine |
| - 17.5 | Valine |
| - 14.0 | Isoleucine |
| - 28.0 | Leucine |
| - 3.5 | Methionine |
| - 14.0 | Phenylalanine |
| - 10.5 | Tyrosine |
| - 7.0 | Tryptophan |
| 1.0 | *Nannochloropsis* chloroplast protein |

*Nannochloropsis* membrane lipids

The ratios between different fatty acids in different membrane lipids were taken from Vieler et al. 2012. Stoichiometric coefficients were set so a metabolite pool consisting of a 100 molecules is produced. The ratio between MGDG, DGDG and SQDG in the cell membrane was taken from Li et al. 2014.

|  | MGDG | DGDG | SQDG |
| --- | --- | --- | --- |
| C14:0 | -14 | -7 | -6 |
| C16:0 | -15 | -36 | -52 |
| C16:1 | -18 | -34 | -41 |
| C18:0 | -2 |  |  |
| C18:1 | -1 | -2 |  |
| C18:2 | -1 | -2 |  |
| C20:4 | -2 |  |  |
| C20:5 | -47 | -19 | -1 |
| Lipid pool | 1 | 1 | 1 |

| *Stoichiometric coefficient* | *Fatty acid pool* |
| --- | --- |
| - 0.27 | MGDG |
| - 0.25 | DGDG |
| - 1.00 | SQDG |
| 1.00 | *Nannochloropsis* membrane lipid fraction |

*Nannochloropsis* pigments

The pigment composition in *Nannochloropsis* was found in Lubián et al. 2000.

| *Stoichiometric coefficient* | *Pigment* |
| --- | --- |
| - 92.6 | Chlorophyll *a* |
| - 1.0 | β-carotene |
| -37.4 | Violaxanthin |
| -1.0 | Zeaxanthin |
| -3.5 | Antheraxanthin |
| -4.5 | Canthaxanthin |
| -6.7 | Astaxanthin |
| 1.0 | *Nannochloropsis* pigment fraction |

***Chlamydomonas***

*Chlamydomonas* chloroplast proteins

The amino acid composition for *Chlamydomonas* chloroplast proteins were taken from Boyle & Morgan, 2009, and stoichiometric coefficients were set so the chloroplast model can create proteins consisting of 350 amino acids.

| *Stoichiometric coefficient* | *Amino acid* |
| --- | --- |
| -19.5 | Aspartic acid |
| - 23.0 | Glutamic acid |
| - 19.0 | Asparagine |
| - 23.0 | Glutamine |
| - 6.0 | Serine |
| - 24.0 | Threonine |
| - 43.0 | Arginine |
| - 1.0 | Histidine |
| - 5.0 | Lysine |
| - 30.0 | Glycine |
| - 1.0 | Cysteine |
| - 13.5 | Proline |
| - 79.0 | Alanine |
| - 17.0 | Valine |
| - 9.0 | Isoleucine |
| - 24.0 | Leucine |
| - 1.0 | Methionine |
| - 10.0 | Phenylalanine |
| - 1.0 | Tyrosine |
| - 1.0 | Tryptophan |
| 1.0 | *Chlamydomonas* chloroplast protein |

*Chlamydomonas* membrane lipids

The ratios between different fatty acids in the different classes of membrane lipids in *Chlamydomonas* were taken from Suh et al. 2015. For *Chlamydomonas*, specific ratios for MGDG, DGDG and SQDG were not found, only the ratio for membrane lipids in general. The same set of stoichiometric coefficients was therefore used for all three lipid classes. Stoichiometric coefficients were set so a metabolite pool consisting of a 100 molecules is produced. The ratio between the different lipid classes was taken from Boudière et al. 2014.

|  | MGDG | DGDG | SQDG |
| --- | --- | --- | --- |
| C14:0 | - 1 | - 1 | - 1 |
| C16:0 | - 25 | - 25 | - 25 |
| C16:1 | - 2 | - 2 | - 2 |
| C18:0 | - 14 | - 14 | - 14 |
| C18:1 | - 3 | - 3 | - 3 |
| C18:2 | - 3 | - 3 | - 3 |
| C18:3 | - 27 | - 27 | - 27 |
| C20:4 | - 1 | - 1 | - 1 |
| C20:5 | - 20 | - 20 | - 20 |
| C22:6 | - 4 | - 4 | - 4 |
| Lipid pool | 1 | 1 | 1 |

| *Stoichiometric coefficient* | *Fatty acid pool* |
| --- | --- |
| - 1.00 | MGDG |
| - 0.36 | DGDG |
| - 0.24 | SQDG |
| 1.00 | *Chlamydomonas* membrane lipid fraction |

*Chlamydomonas* pigments

The pigment composition in *Chlamydomonas* was found in Eichenberger et al. 1986.

| *Stoichiometric coefficient* | *Pigment* |
| --- | --- |
| - 100.00 | Chlorophyll *a* |
| - 49.68 | Chlorophyll *b* |
| - 0.85 | α-carotene |
| - 13.17 | β-carotene |
| - 9.25 | Violaxanthin |
| - 7.21 | Neoxanthin |
| - 16.69 | Lutein |
| 1.0 | *Chlamydomonas* pigment fraction |

***Phaeodactylum***

*Phaeodactylum* chloroplast proteins

The amino acid composition for *Phaeodactylum* chloroplast proteins were taken from Brown, 1991, and stoichiometric coefficients were set so the chloroplast model can create proteins consisting of 350 amino acids.

| *Stoichiometric coefficient* | *Amino acid* |
| --- | --- |
| - 28.0 | Aspartic acid |
| - 35.0 | Glutamic acid |
| - 3.5 | Asparagine |
| - 3.5 | Glutamine |
| - 21.0 | Serine |
| - 17.5 | Threonine |
| - 24.5 | Arginine |
| - 7.0 | Histidine |
| - 21.0 | Lysine |
| - 21.0 | Glycine |
| - 3.5 | Cysteine |
| - 21.0 | Proline |
| - 24.5 | Alanine |
| - 21.0 | Valine |
| - 17.5 | Isoleucine |
| - 28.0 | Leucine |
| - 7.0 | Methionine |
| - 24.5 | Phenylalanine |
| - 14.0 | Tyrosine |
| - 7.0 | Tryptophan |
| 1.0 | *Phaeodactylum* chloroplast protein |

*Phaeodactylum* membrane lipids

The ratios between different fatty acids in the different classes of membrane lipids in *Phaeodactylum* were taken from Tonon et al. 2002. For *Phaeodactylum*, specific ratios for MGDG, DGDG and SQDG were not found, only the ratio for membrane lipids in general. The same set of stoichiometric coefficients was therefore used for all three lipid classes. Stoichiometric coefficients were set so a metabolite pool consisting of a 100 molecules is produced. The ratio between different membrane lipids was found in Arao et al. 1987.

|  | MGDG | DGDG | SQDG |
| --- | --- | --- | --- |
| C14:0 | - 12 | - 12 | - 12 |
| C16:0 | - 14 | - 14 | - 14 |
| C16:1 | - 31 | - 31 | - 31 |
| C18:0 | - 2 | - 2 | - 2 |
| C18:1 | - 6 | - 6 | - 6 |
| C20:5 | - 31 | - 31 | - 31 |
| C22:6 | - 4 | - 4 | - 4 |
| Lipid pool | 1 | 1 | 1 |

| *Stoichiometric coefficient* | *Fatty acid pool* |
| --- | --- |
| - 1.00 | MGDG |
| - 0.30 | DGDG |
| - 0.34 | SQDG |
| 1.00 | *Phaeodactylum* membrane lipid fraction |

*Phaeodactylum* pigments

*Phaeodactylum* contains both fucoxanthin, diatoxanthin, diadinoxanthin and chlorophyll *c* in addition to chlorophyll *a* (Rebolloso-Fuentes et al. 2001). The production of these pigments requires specific pathways that have not yet been added to the chloroplast model. The pigment pool of *Phaeodactylum* therefore so far only consists of chlorophyll *a*.

| *Stoichiometric coefficient* | *Pigment* |
| --- | --- |
| - 100.00 | Chlorophyll *a* |
| 1.0 | *Phaeodactylum* pigment fraction |
